# Supplementary material for: Empirical methods for controlling false positives and estimating confidence in ChIP-Seq peaks
Source: BMC Bioinformatics. 2008 Dec 5;9:523. doi: 10.1186/1471-2105-9-523 (PMC2628906; doi:10.1186/1471-2105-9-523)
Supplement: Additional File 1 — A variety of html documents from the USeq web site detailing the available applications, their best usage, output file type descriptions, command line menus, etc. [file 1471-2105-9-523-S1.zip › USeqUserGuides/JavaDoc.zip!/JavaDoc/bioroot/plasmid/PlasmidBase.html]

PlasmidBase


|  |  |  |
| --- | --- | --- |
| **Overview** \| **Package** \| **Class** \| **Use** \| **Tree** \| **Deprecated** \| **Index** \| **Help** | |  |
| **PREV CLASS**   **NEXT CLASS** | **FRAMES**    **NO FRAMES**     **All Classes** |  |
| SUMMARY: NESTED \| FIELD \| CONSTR \| METHOD | DETAIL: FIELD \| CONSTR \| METHOD |  |


---


## bioroot.plasmid Class PlasmidBase

```
java.lang.Object
  javax.servlet.GenericServlet
      javax.servlet.http.HttpServlet
          bioroot.plasmid.PlasmidBase
```

**All Implemented Interfaces:**: java.io.Serializable, javax.servlet.Servlet, javax.servlet.ServletConfig

---

``` public class PlasmidBase extends javax.servlet.http.HttpServlet ```

**Author:**
:   nix
    Contains static methods for Plasmids

**See Also:**: Serialized Form

---

| **Field Summary** | |
| --- | --- |
| `static java.lang.String[]` | `searchHeadings`             Use to make a hash of column heading in which to search, name then the actual search column name. |
| `static java.lang.String[]` | `stringFeatures`             Use to make a default plasmid Feature[]. |


**Constructor Summary**

`PlasmidBase()`


| **Method Summary** | |
| --- | --- |
| `void` | `doGet(javax.servlet.http.HttpServletRequest request, javax.servlet.http.HttpServletResponse response)` |
| `void` | `doPost(javax.servlet.http.HttpServletRequest request, javax.servlet.http.HttpServletResponse response)` |
| `static void` | `findOligoMatches(UserBean user, PlasmidBean[] plasmids, DBUtil bioRoot)`             Uses Blast to find oligo matches to a given array of plasmid from all the oligos in a labgroup visible to the user. |
| `static void` | `findOligos(UserBean user, PlasmidBean[] plasmids, DBUtil bioRoot)`             Uses Blast to find oligo matches to a given array of plasmid from all the oligos in a labgroup visible to the user. |
| `static void` | `findOligos(UserBean user, PlasmidBean plasmid, DBUtil bioRoot)`             Uses Blast to find oligo matches to a plasmid from all the oligos in a labgroup visible to the user. |
| `static java.lang.String` | `getPlasmidForm(PlasmidBean ob, DBUtil bioRoot, UserBean userBean)`             Returns a plasmid form filled with the values in the bean. |
| `static java.lang.String` | `getPlasmidReport(PlasmidBean pb, DBUtil bioRoot, UserBean userBean, boolean commonForm)`             Returns an plasmid report. |
| `static java.lang.String[]` | `getSearchColumnNames()`             Returns a list of the search column names used in an SQL statment , ie Oligo.name, 1/2 the searchHash. |
| `static java.util.LinkedHashMap` | `getSearchHash()` |
| `static java.lang.String[]` | `getSearchNames()` |
| `static java.lang.String` | `makeOligoConcat(BlastMatcher[] oligos)`             Converts a BlastMatcher[] to a concat of oligo sequences and NNNNNNNNNN spacers. |
| `static PlasmidBean[]` | `reversePlasmidBeans(PlasmidBean[] beans)`             Reverses the order of an array of PlasmidBeans. |

**Methods inherited from class javax.servlet.http.HttpServlet**

`service`

**Methods inherited from class javax.servlet.GenericServlet**

`destroy, getInitParameter, getInitParameterNames, getServletConfig, getServletContext, getServletInfo, getServletName, init, init, log, log`

**Methods inherited from class java.lang.Object**

`equals, getClass, hashCode, notify, notifyAll, toString, wait, wait, wait`

**Field Detail**

### stringFeatures

```
public static java.lang.String[] stringFeatures
```

:   Use to make a default plasmid Feature[]. Watch order, stops when it finds a false.

---


### searchHeadings

```
public static final java.lang.String[] searchHeadings
```

:   Use to make a hash of column heading in which to search, name then the actual search column name.


**Constructor Detail**

### PlasmidBase

```
public PlasmidBase()
```


**Method Detail**

### getSearchHash

```
public static java.util.LinkedHashMap getSearchHash()
```

---


### getSearchNames

```
public static java.lang.String[] getSearchNames()
```

---


### getSearchColumnNames

```
public static java.lang.String[] getSearchColumnNames()
```

:   Returns a list of the search column names used in an SQL statment , ie Oligo.name, 1/2 the searchHash.

---


### doGet

```
public void doGet(javax.servlet.http.HttpServletRequest request,
                  javax.servlet.http.HttpServletResponse response)
           throws javax.servlet.ServletException,
                  java.io.IOException
```

:   **Overrides:**: `doGet` in class `javax.servlet.http.HttpServlet`
:   **Throws:**: `javax.servlet.ServletException`: `java.io.IOException`

---


### doPost

```
public void doPost(javax.servlet.http.HttpServletRequest request,
                   javax.servlet.http.HttpServletResponse response)
            throws javax.servlet.ServletException,
                   java.io.IOException
```

:   **Overrides:**: `doPost` in class `javax.servlet.http.HttpServlet`
:   **Throws:**: `javax.servlet.ServletException`: `java.io.IOException`

---


### reversePlasmidBeans

```
public static PlasmidBean[] reversePlasmidBeans(PlasmidBean[] beans)
```

:   Reverses the order of an array of PlasmidBeans.

---


### getPlasmidReport

```
public static java.lang.String getPlasmidReport(PlasmidBean pb,
                                                DBUtil bioRoot,
                                                UserBean userBean,
                                                boolean commonForm)
```

:   Returns an plasmid report.

---


### getPlasmidForm

```
public static java.lang.String getPlasmidForm(PlasmidBean ob,
                                              DBUtil bioRoot,
                                              UserBean userBean)
```

:   Returns a plasmid form filled with the values in the bean. Must add a form entry. ie
    + "

    " and start and stop HTML stuff

---


### findOligos

```
public static void findOligos(UserBean user,
                              PlasmidBean plasmid,
                              DBUtil bioRoot)
```

:   Uses Blast to find oligo matches to a plasmid from all the oligos in a labgroup visible to the user.
    Will only attempt a BLAST if PlasmidBean.blasted == false.

---


### findOligos

```
public static void findOligos(UserBean user,
                              PlasmidBean[] plasmids,
                              DBUtil bioRoot)
```

:   Uses Blast to find oligo matches to a given array of plasmid from all the oligos in a labgroup visible to the user.
    Will not search if PlasmidBean.blasted = true.

---


### findOligoMatches

```
public static void findOligoMatches(UserBean user,
                                    PlasmidBean[] plasmids,
                                    DBUtil bioRoot)
```

:   Uses Blast to find oligo matches to a given array of plasmid from all the oligos in a labgroup visible to the user.
    Will search everything irregardles of PlasmidBean.blasted flag.
    Avoid calling this method directly. Use findOligos method instead!

---


### makeOligoConcat

```
public static java.lang.String makeOligoConcat(BlastMatcher[] oligos)
```

:   Converts a BlastMatcher[] to a concat of oligo sequences and NNNNNNNNNN spacers.
    Updates array with bp coordinates for positions of the oligo in the concat.


---


|  |  |  |
| --- | --- | --- |
| **Overview** \| **Package** \| **Class** \| **Use** \| **Tree** \| **Deprecated** \| **Index** \| **Help** | |  |
| **PREV CLASS**   **NEXT CLASS** | **FRAMES**    **NO FRAMES**     **All Classes** |  |
| SUMMARY: NESTED \| FIELD \| CONSTR \| METHOD | DETAIL: FIELD \| CONSTR \| METHOD |  |


---
